# A case–control study to evaluate the impact of the breast screening programme on breast cancer incidence in England

**DOI:** 10.1002/cam4.5004

**Published:** 2022-07-18

**Authors:** Oleg Blyuss, Amanda Dibden, Nathalie J. Massat, Dharmishta Parmar, Jack Cuzick, Stephen W. Duffy, Peter Sasieni

**Affiliations:** ^1^ Centre for Prevention, Detection, and Diagnosis, Wolfson Institute of Population Health Queen Mary University of London London UK; ^2^ School of Cancer and Pharmaceutical Sciences, Faculty of Life Sciences and Medicine King's College London London UK

**Keywords:** breast cancer, cancer prevention, screening, women's cancer

## Abstract

**Background:**

There is uncertainty about overdiagnosis in mammography screening.

**Methods:**

We aimed to estimate the effect of screening on breast cancer incidence and overdiagnosis in the NHS Breast Screening Programme in England. The study included 57,493 cases and 105,653 controls, with cases defined as women diagnosed at ages 47–89 with primary breast cancer, invasive or ductal carcinoma in situ, in 2010 or 2011. Where possible, two controls were selected per case, matched on date of birth and screening area.

Conditional logistic regression was used to estimate the effect of screening on breast cancer risk, with adjustment for potential self‐selection bias. Results were combined with national incidence data to estimate absolute rates of overdiagnosis. Overdiagnosis was calculated as the cumulative excess of cancers diagnosed in the age group 50–77 in a woman attending three‐yearly screening between ages 50 and 70 compared with a woman attending no screens.

**Results:**

The estimated number of cases overdiagnosed in women attending all screens in the programme was 679.3 per 100,000 without adjustment for self‐selection bias and 261.2 per 100,000 with adjustment. These corresponded to an estimated 9.5% of screen‐detected cancers overdiagnosed without adjustment and 3.7% with adjustment for self‐selection.

**Conclusions:**

The NHS Breast Screening Programme in England confers at worst modest levels of overdiagnosis.

## Graphical abstract

We calculated estimates of relative risk of breast cancer diagnosis associated with screening exposure. We combined these with national incidence figures to obtain estimates of overdiagnosis associated with breast cancer screening. We found at worst modest levels of overdiagnosis as a result of the breast screening programme in England.

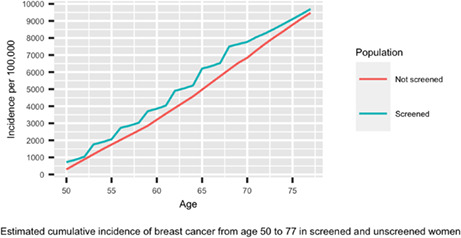

## 1 INTRODUCTION

In the UK, breast cancer accounts for 31% of all new female cancers diagnosed each year, with breast cancer incidence increasing by 4% in the last decade in the UK, and prior to that increasing dramatically worldwide in the latter half of the twentieth century.^1^, ^2^ Despite the continued rise in breast cancer incidence, Mortality from breast cancer is declining.^3^ This decline in mortality is likely due to a combination of the availability of evolving and improved treatments as well as earlier diagnosis, facilitated in large measure by mammography screening. The National Health Service Breast Screening Programme (NHSBSP) was initiated in 1988. The NHSBSP currently invites aged 50–70 to three‐yearly screening, with two‐view mammography.

There remains debate about the potential harms of screening, notably overdiagnosis. Overdiagnosis is usually defined as the detection of cancer through screening that would not have been detected in a woman's lifetime in the absence of screening.^4^ The UK Independent Breast Screening Review estimated overdiagnosis from randomised controlled trials (RCT) of screening, as a proportion of cancers diagnosed in a population invited to screening, to be 11%.^5^ In organised mammography screening programmes, there is a large variation in estimated rates of overdiagnosis^5^, ^6^, ^7^, ^8^ This large variation is partly due to the differing methods used to calculate overdiagnosis, for example, whether DCIS cancers are included, whether there is a sufficient follow‐up period after cessation of screening, the method used to adjust for lead time (the interval between detection by screening and the time the cancer would have been diagnosed in the absence of screening), and which denominator is used.^4^, ^5^

Until now, most studies estimating overdiagnosis have been ecological or cohort studies. Case–control studies nested within a cohort population also allow for overdiagnosis rates to be robustly estimated^9^ provided that underlying risk differences between screened and unscreened women can be accounted for. Here we present results from one of a series of case–control evaluations which are being conducted to evaluate the NHSBSP in England.^10^ The aim of this case–control study is to estimate the relative effect of attendance at screening on breast cancer incidence and to use national incidence data to give an estimate of the absolute level of overdiagnosis specific to the NHSBSP in England.

## 2 METHODS

Our estimation of overdiagnosis was a multi‐stage process, as follows:

1. Conduct a case–control study where cases were women with breast cancer and controls were women with no diagnosis of breast cancer prior to the age of their individually matched case (although in principle, they could develop breast cancer thereafter and potentially be a case), with aspects of screening history as the potential predictor variables. This was to estimate the effect of being screened on risk of being diagnosed with breast cancer. The controls were matched with cases on date of birth and screening area, to ensure a level of comparability with respect to the opportunity to be screened.
2. Only screening before the diagnosis date of the cases (for both cases and their matched controls) was considered, again to ensure comparability with respect to the opportunity to be screened.
3. Women who choose to be screened may have different underlying incidence rates of breast cancer a priori than women who choose not to be screened, for a number of reasons, including higher socioeconomic status, other breast cancer risk factors, and greater breast awareness. We therefore, adjusted the estimated effect in one above for self‐selection bias.
4. The odds ratio estimates of relative risk derived from 1 to 3 above give the relative effect of screening on incidence. To obtain the absolute effects, we then combined our estimated relative risk of diagnosis of breast cancer for screened women versus unscreened with national incidence rates of breast cancer, to estimate the long‐term incidence of breast cancer in a screened versus unscreened cohort, to 7 years beyond the upper age limit for screening.
5. The excess incidence in a screened population for the screening ages plus 7 years after the upper age limit was regarded as an estimate of overdiagnosis associated with screening. Since only screen‐detected cancers can be overdiagnosed, we divided this absolute excess by numbers screened to give the risk of overdiagnosis in persons attending for screening, and by the number of screen‐detected cancers to give the percentage of screen‐detected cancers which were estimated to be overdiagnosed.

### 2.1 Definition of cases and controls

Cases were women who were diagnosed with primary breast cancer (invasive or in situ) in 2010 or 2011 and aged between 47 and 89 years. Cases were not selected on the basis of mode of detection or screening exposure, since in the first instance we wished to estimate the effect of screening on overall incidence. We chose a lower age limit of 47 because substantial numbers of women have been offered screening from age 47 rather than 50 in the UK due to an ongoing trial. The upper age limit of 89 years was chosen as the screening programme in England has been going on for decades, and women in their late 80’s in 2011 could have been screened in their 60’s 15–20 years earlier. Cases may have died by the end of the study period. Controls were women sampled from the general population of those invited for screening and alive at the time of their corresponding case's date of diagnosis, matched on date of birth (within 1 month) and screening area at date of their case's diagnosis. Controls may have been diagnosed with breast cancer, but not before their case's date of diagnosis. Where possible, two controls were selected per case. Controls were assigned a pseudodiagnosis date equal to the date of diagnosis of their matched case. Both cases and controls must have received at least one invitation to screening prior to the diagnosis/pseudodiagnosis date. Controls must have received their first invitation to screening within 4 years of their matched case's date of first invitation.

### 2.2 Data extraction

Cases were identified from the National Cancer Registration and Analysis Service (NCRAS) database accessed through the Office for Data Release of Public Health England (PHE).

NHS Digital used the National Health Application and Infrastructure Services (NHAIS) system to identify two matched controls per case and provided breast screening histories. We excluded screens occurring outside the usual call/recall system of the national screening programme. The entire screening histories of the study subjects were considered up to and including their date of diagnosis/pseudodiagnosis.

Age‐specific invasive cancer incidence rates for England in 2011 were obtained from public domain data from the Office for National Statistics.^11^

### 2.3 Sample size

Previous results suggested that 81.7% of controls will have attended at least one screen.^12^ If we assume 80% concordance between cases and controls, and that 82.3% of cases would ever have been screened (a 6% increase in odds of cases having been screened), 43,000 cases and the same number of matched controls would confer 90% power to detect this small difference as significant (5% significance level, two‐sided testing). Our sampling plan above yielded 57,493 cases and 105,653 controls.

### 2.4 Statistical methods

#### 2.4.1 Regression modelling

Conditional logistic regression was used to estimate the effect of attendance at screening on the chance of being diagnosed with breast cancer. We estimated both the effect of ever attending breast screening on breast cancer incidence and the association between breast cancer incidence and time between last screen and diagnosis/pseudodiagnosis.

#### 2.4.2 Adjustment for self‐selection bias

Women who attend screening have been observed to have higher underlying Incidence of breast cancer than those who do not,^13^ although they may have lower mortality from the disease.^10^ This may have a number of mechanisms of action, one being higher socioeconomic status or other risk factors, another being a greater tendency to breast awareness among women who choose to accept the offer of screening. To adjust for this self‐selection, we considered the effect of screening exposure on incidence of breast cancer by time since last screen. The classic pattern of this effect is an increase in incidence in the year following a screen, due to screen‐detected cancers, followed by a deficit, with incidence gradually approaching that in unscreened women over 5–10 years.^9^ If, at the end of that period, the risk of diagnosis of breast cancer among screened women not only reaches the unscreened risk but rises above it, this must represent an underlying difference between those choosing to be screened and those choosing not to be screened. This excess must therefore be the effect of self‐selection since screening cannot increase the incidence 10 years later. We therefore, divided the estimated odds ratios (OR) associated with screening attendance by the OR associated with last screen 10 or more years ago. This assumes that the same correction is appropriate for all years since last screen.

#### 2.4.3 Calculation of overdiagnosis

Overdiagnosis was calculated as the estimated cumulative excess of cancers diagnosed in the age group 50–77 in a woman attending three‐yearly screening between ages 50 and 70 (since this is the screening regimen offered by the NHSBSP) compared with a woman attending no screens. As noted above, we estimated the OR estimates of relative risk of being diagnosed with breast cancer by time since last screen. To estimate overdiagnosis in the NHSBSP, we combined these with national incidence rates to estimate the cumulative incidence of breast cancer to age 77 in a woman attending all seven three‐yearly screens between ages 50 and 70 and a woman not attending screening during this period of life. We used age 77 as the upper age limit as this would add around double the frequently estimated mean sojourn time of between 3 and 4 years.^8^ From published figures, we had the overall incidence of breast cancer by 5 year age groups for the year 2011.^11^ In the NHSBSP, attendance at screening varies but is approximately 70% on average.^14^ For a given year within a 3‐year screening cycle, therefore, we have:

$$I=0.3\times I_{0}+0.7\times \bar{\mathrm{RR}}\times I_{0}$$

where *I* is the published overall incidence for that 3‐year period of age, applied to a cohort of 100,000 women alive at age 50 and attenuated for each year of age thereafter by the all‐cause death rate in 2010–2012 for females of that age in the UK.^15^ *I*_*0*_ is the corresponding average incidence for the relevant year of age in an unscreened population and $\bar{\mathrm{RR}}$ the average OR estimate of relative risk for that 3‐year period. Since *I* is known and

$$\bar{\mathrm{RR}}=\left({\text{OR}}_{1}+{\text{OR}}_{2}+{\text{OR}}_{3}\right)/3$$

is calculated as the average of the age‐specific ORs for the first, second and third years after a screen, we could derive *I*_*0*_ from the first equation above. The incidence in a screened population in a given year after the screening was estimated as *I*_*0*_ multiplied by the OR for that individual year (whereas in an unscreened population, incidence will change only slowly and smoothly, one expects substantial differences in incidence between individual years since screen in a screened population). An example of the detailed calculations is given in the Supplementary Material. This is illustrated in the Results section. We then compared the total incidence in screened and unscreened populations to estimate overdiagnosis.

We derived two estimates of overdiagnosis and expressed each in three ways. First, using the ORs adjusted for self‐selection, we estimated the absolute risk of overdiagnosis associated with offering screening (with an attendance rate of 70%), the absolute risk in a woman attending screening, and finally, the estimated percentage of screen‐detected cancers overdiagnosed. We also estimated the same quantities without adjustment for self‐selection.

Analyses were conducted using R Statistical Software version 3.6.2^16^ and Stata version 16^17^7.

## 3 RESULTS

**FIGURE 1:**
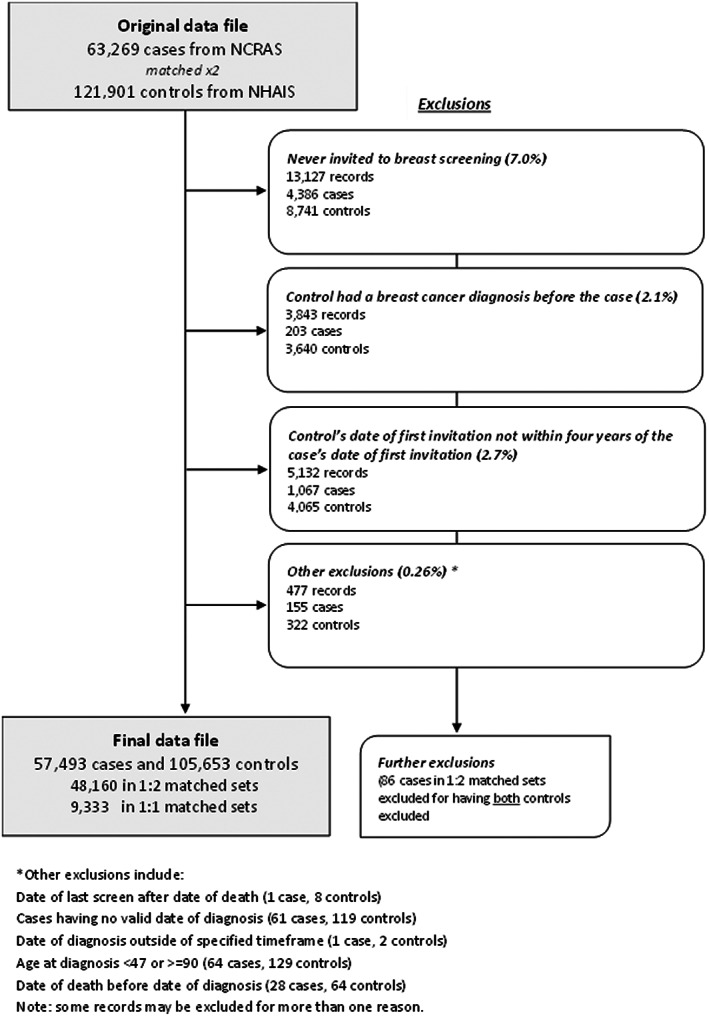
Study flow diagram

We identified a total of 63,269 cases and 121,901 controls. However, 22,024 women (5776 cases and 16,248 controls, 12% of the study population) were excluded as they did not meet one or more of the eligibility criteria (Figure 1). The final dataset contained records of 57,493 cases and 105,653 controls, made up of 48,160 1:2 matched sets and 9333 1:1 matched sets.

Table 1 shows the demographics and screening histories of the women included. The average age at diagnosis was 64.2 for cases and 64.0 for controls. The median age at first screen was 51.3 for cases and 51.2 for controls, with cases attending slightly more screens on average than controls, 3.43 and 3.33 respectively. The median age at last screen was 62.4 for cases and 62.0 for controls. Approximately 54% of cancers were diagnosed when women were under the age of 65.

**TABLE 1:** Patient demographics and screening history by case–control status

	Cases (*n* = 57,493)	Controls (*n* = 105,653)
Breast cancer diagnosis		
Age at first diagnosis/ pseudodiagnosis		
47–54	10,403 (18.1)	19,928 (18.9)
55–59	8730 (15.2)	16,209 (15.3)
60–64	11,709 (20.4)	21,441 (20.3)
65–69	10,485 (18.2)	18,763 (17.8)
70+	16,166 (28.1)	29,312 (27.7)
Median age at first diagnosis/pseudodiagnosis in years (range)	64.2 (47.2–89.9)	64.0 (47.2–90.0)
Breast screening history		
Number of screening invitations		
1	10,199 (17.7)	20,866 (19.7)
2	8185 (14.2)	15,241 (14.4)
3	7507 (13.1)	14,441 (13.7)
4	7992 (13.9)	15,054 (14.2)
5+	23,610 (41.1)	40,051 (37.9)
Median age at first screening invitation in years (range)	51.3 (47.0–73.0)	51.2 (47–73)
Median age at last screening invitation in years (range)	62.4 (47.0–73.8)	62 (47–74)
Number of screens (Count, %)		
Never screened	5814 (10.1)	12,633 (12.0)
1	11,626 (20.1)	21,745 (20.6)
2+	40,053 (69.7)	71,275 (67.4)
Mean number of screening rounds (excluding never screened) (range)	3.4 (1.0–13.0)	3.3 (1.0–12.0)

There was a significant 16% increase in diagnosis of breast cancer in women who attended at least one screening invitation after adjustment for self‐selection (Table 2), with an OR of 1.16 (95% CI 1.09–1.23, *p* < 0.001). From Table 3, the OR associated with screening more than 9 years prior to diagnosis/pseudodiagnosis was 1.05 (95% CI 0.99–1.11, *p* = 0.08). This was therefore used as the self‐selection correction factor.

**TABLE 2:** Conditional logistic regression evaluating the association between screening attendance and breast cancer diagnosis

Exposure	Controls/cases	OR (95% CI)	OR (95% CI) corrected for self‐selection^a^
Never screened	12,633/5814	1.00 (−)	1.00 (−)
Ever screened	93,020/51,679	1.22 (1.18–1.26)	1.16 (1.09–1.23)

a The self‐selection correction factor was estimated to be 1.05.

**TABLE 3:** Conditional logistic regression evaluating the association between time since last screen attendance and breast cancer diagnosis

Time between last screen and diagnosis/pseudodiagnosis	Controls/cases	OR (95% CI)	OR (95% CI) corrected for self‐selection^a^
Never screened	12,633/5814	1.00 (−)	1.00(−)
<1 year	25,791/27,368	2.46 (2.36–2.56)	2.34 (2.21–2.48)
1–2 years	20,216/4160	0.42 (0.39–0.44)	0.40 (0.37–0.42)
2–3 years	17,943/4841	0.54 (0.51–0.57)	0.51 (0.48–0.55)
3–4 years	3611/1723	0.85 (0.79–0.92)	0.81 (0.75–0.88)
4–5 years	3079/1392	0.77 (0.71–0.83)	0.73 (0.67–0.79)
5–6 years	2832/1393	0.88 (0.82–0.96)	0.84 (0.77–0.92)
6–7 years	1630/877	1.01 (0.91–1.11)	0.96 (0.87–1.06)
7–8 years	1050/559	0.99 (0.87–1.11)	0.94 (0.83–1.06)
8–9 years	808/451	1.04 (0.91–1.18)	0.99 (0.87–1.13)
>9 years	16,060/8915	1.05 (0.99–1.11)	1.00 (−)

a The self‐selection correction factor was estimated to be 1.05.

Considering time since last screen (Table 3), there was a substantial increase in odds of breast cancer diagnosis within 1 year of last screen (adjusted OR 2.34, 95% CI 2.21–2.48). This is comprised mainly of screen‐detected cancers. After this initial increase, incidence decreased with a 60% reduction in women who were last screened in the second year following the last screen. The decrease in incidence was then attenuated over time, returning to the unscreened incidence after around 7 years, and going slightly above the unscreened incidence at 9+ years, suggesting a modest amount of self‐selection bias (OR = 1.05 as noted above). Odds ratios corresponding to those in Table 3 but specific to age groups 47–59, 60–64, 65–69, and 70+ are given in Table S1.

Table 4 shows the total invasive and in situ breast cancer incidence in the population in 2011 by age, published by the Office for National Statistics,^11^ attenuated annually by all‐cause mortality in each year of age, and the corresponding estimated cancer incidence in cohorts screened and not screened from age 50, calculated using the age‐specific ORs estimated in this study (Table S1). These were then used to estimate the cumulative incidence to age 77 in screened and unscreened women, and therefore the absolute rate of overdiagnosis.

**TABLE 4:** Estimated breast cancer incidence per 100,000 by individual year of age in screened and non‐screened populations, from age 50 to 77

Age	Screening episode	Total incidence	Cases per 100,000 correcting for annual mortality	OR	OR corrected for self‐selection	Incidence in a population not screened	Adjusted incidence in a population not screened^a^	Incidence in a screened population	Adjusted incidence in a screened population^a^
50	X	332.8	332.8	2.55	2.43	287.2	297.4	732.4	722.7
51		332.8	332.1	0.50	0.48	286.6	296.8	143.3	142.4
52		332.8	331.3	0.63	0.60	285.9	296.0	180.1	177.6
53	X	332.8	330.4	2.55	2.43	285.1	295.2	727.1	717.4
54		332.8	329.4	0.50	0.48	284.3	294.4	142.1	141.3
55		312.7	308.5	0.63	0.60	266.3	275.7	167.7	165.4
56	X	312.7	307.4	2.55	2.43	265.3	274.7	676.6	667.6
57		312.7	306.3	0.50	0.48	264.3	273.7	132.2	131.4
58		312.7	305.0	0.63	0.60	263.2	272.6	165.8	163.5
59	X	312.7	303.6	2.55	2.43	270.2	280.1	689.0	680.6
60		396.0	382.6	0.43	0.41	340.5	352.9	146.4	144.7
61		396.0	380.5	0.55	0.52	338.6	351.0	186.2	182.5
62	X	396.0	378.2	2.68	2.55	327.8	340.1	878.4	867.4
63		396.0	375.8	0.43	0.41	325.7	338.0	140.0	138.6
64		396.0	373.3	0.55	0.52	323.5	335.7	177.9	174.6
65	X	457.0	427.6	2.60	2.48	385.3	398.7	1001.8	988.8
66		457.0	424.1	0.37	0.35	382.2	395.5	141.4	138.4
67		457.0	420.2	0.50	0.48	378.6	391.8	189.3	188.1
68	X	457.0	415.9	2.60	2.48	378.0	391.3	982.8	970.4
69		457.0	411.4	0.37	0.35	373.9	387.0	138.3	135.5
70		363.5	323.2	0.46	0.44	293.7	304.1	135.1	133.8
71		363.5	318.7	0.68	0.65	413.2	426.1	281.0	277.0
72		363.5	313.9	0.59	0.56	389.3	402.2	229.7	225.2
73		363.5	308.6	0.75	0.71	360.8	373.1	270.6	264.9
74		363.5	303.0	0.83	0.79	344.8	356.2	286.2	281.4
75		402.5	328.6	0.80	0.76	364.3	375.9	291.4	285.7
76		402.5	321.1	0.85	0.81	347.9	358.8	295.7	290.6
77		402.5	312.8	0.93	0.89	328.9	338.9	305.9	301.6
Total		10,517.5	9706.2	‐	‐	9155.5	9473.8	9834.8	9699.0

a Adjusted for self‐selection bias.

The estimated incidence in the invited population was more than double that of the unscreened population at the individual years of age when the screens take place (50, 53, 56 etc.). This then fell to approximately half the rate of the unscreened population in the intermittent years. During the screening years, the estimated rate of cancer steadily increased with the above noted peaks every 3 years in the screened population, until age 68, the age at which women attend their last screen. Peaks are particularly high, as the interscreening interval is 3 years. Smaller peaks would be observed at greater frequency if the interval were shorter. It then fell below the rate of the unscreened population in the subsequent years, gradually returning to the rate in the unscreened population over the 9 years to age 77. Following adjustment for self‐selection, the excess cumulative incidence in the screened population was 261.2 per 100,000 (9699.0 − 9437.8), with 95% CI (55.9–1221.3). Overdiagnosis was therefore estimated as just under 3 overdiagnosed cancers per 1000 women attending screening throughout the programme. This corresponds to 182.4 per 100,000 invited, just under two overdiagnosed cancers per 1000 women invited, and around 3% (261.2/9699.0) of cancers diagnosed in women attending for screening estimated as overdiagnosed. In women attending for screening in the NHSBSP, around 8 per 1000 have a cancer detected at screening in a given round and 3 per 1000 are diagnosed with an interval cancer^14^, ^18^ Thus we estimated that the proportion of screen‐detected cancers overdiagnosed is

$$\frac{261.2}{9699.0}\times \frac{11}{8}=0.037$$

That is, we estimate that 3.7% (95% CI 0.8–17.4%) of screen‐detected cancers were overdiagnosed.

With no adjustment for self‐selection, the difference in cumulative incidence to age 77 was 679.3 per 100,000 (9834.8 − 9155.5), with a 95% CI of (367.7–1224.9) suggesting a risk of overdiagnosis of almost 7 per 1000 women attending screening throughout the programme, and 475.5 per 100,000 invited (assuming 70% attendance), or just under 5 per 1000 women invited. This corresponds to around 7% (95% CI of cancers in women attending for screening being overdiagnosed (669.6/10,718.5)). Using the proportion of screen‐detected cancers as above, without adjustment for self‐selection, we would estimate that 9.5% (95% CI 5.2–17.2%) of screen‐detected cancers were overdiagnosed.

## 4 DISCUSSION

We estimated the effect of attendance at screening in the NHSBSP on breast cancer incidence and found that attending at least one screen increases incidence by 16% after adjustment for self‐selection. There was an increase in incidence in the first year after most recent screen, with an adjusted odds ratio of 2.34. This fell to 0.40 in the second year, and gradually increased as time since last screen increased, reaching no difference after around 7 years. Beckmann et al.^9^ saw a similar pattern when assessing incidence by time since last screen, with an odds ratio of 2.35 within the first year following screening, which fell to below one in the second year and tended towards no difference as time since last screen increased. The spike in incidence in the first year since last screen was due to screen‐detected cancers, with 89% of cancers diagnosed in year one being categorised as screen‐detected.

To estimate overdiagnosis associated with the NHSBSP, we calculated the expected cumulative incidence of breast cancer from age 50 to age 77, 7 years after screening ceases to be offered, to accommodate lead time. With adjustment for self‐selection, the absolute number of cancers overdiagnosed associated with invitation to and attendance at screening were estimated as 182.4 and 261.2 per 100,000 women respectively. As a proportion of screen detected cancers, we found overdiagnosis to be approximately 4%. The range of uncertainty on the estimate, as represented by the 95% confidence interval, was relatively wide, from less than 1% to just over 17%. This result is consistent with the proportion of overdiagnosis estimated by Johns et al. in a recent cohort study analysing the effect of screening within the NHSBSP, although their estimate used a different denominator to the one used here.^19^ It is intuitive that there will always be a small amount of overdiagnosis associated with screening, as a proportion of women will inevitably die in the period between when they were screen detected and when their cancer would have been diagnosed symptomatically. A more pessimistic estimate without adjustment for self‐selection suggested that 9–10% of screen‐detected cancers were overdiagnosed. It should be noted that while the proportion of screen‐detected cancers which are overdiagnosed is of interest to the cancer researcher, for the woman deciding whether to accept an offer of screening, the more useful measure is the absolute probability of having a cancer overdiagnosed if she chooses to be screened. We estimated this as either just under 7 per 1000 screened or just under 3 per 1000 screened when self‐selection was adjusted for.

As screening brings forward the diagnosis of cancers in women who attend, the incidence in screened women will be higher than in unscreened women in the screening period but once screening has stopped, the incidence in the screened group will fall to below that in unscreened women unless the entire observed excess is due to overdiagnosis. However, there needs to be sufficient follow‐up after screening has stopped to observe this, with the UK Independent Review concluding that this follow‐up period should be at least 5–10 years.^5^ Our study included individual‐level data with long‐term observation of over 20 years between last screening invitation and breast cancer diagnosis/pseudodiagnosis in some cases, which should be an adequate length of time for any excess from lead time to have disappeared. We considered the excess of cancers up to and including age 77 as a reasonable estimate of overdiagnosis.

Even our more pessimistic estimate of overdiagnosis was lower than suggested in the past. The UK Independent Review estimated overdiagnosis as the difference in incidence between study and control groups in three selected trials of screening and obtained considerably higher estimates.^5^ However, it has been pointed out that there are major methodological problems in using them to estimate overdiagnosis.^20^, ^21^ Zackrisson and colleagues estimated the long‐term excess of breast cancer incidence as 10% in the group allocated to screening, from the Malmö Mammographic Screening Trial, which is similar to our unadjusted estimate.^22^ A subsequent refined analysis from the same trial gave an estimate of 1%, more in the region of our adjusted estimate.^21^

Our estimates of overdiagnosis pertain to all breast cancers, invasive and in situ. Estimation separately for invasive and non‐invasive cancers is a target for the future. It may be that inclusion of in situ cancers could engender a bias towards overestimation of overdiagnosis, since longer such cases might have lead times in excess of 10 years. However, the odds of diagnosis of invasive or in situ cancer equalised between screened and unscreened subjects around 7 years after the last screen.

It should also be noted that although all invasive cases were first invasive primary cancers of the breast, some may have had a previous in situ breast cancer before age 47 (albeit very few). Since none of the controls had previous invasive or in situ cancer, this might also lead to overestimation of overdiagnosis if it led to those few cases to be more likely to adhere to screening.

These results are based on cancers diagnosed in 2010 and 2011. As the programme itself has not changed radically since then, one would expect overdiagnosis rates per person screened and per screen‐detected cancer to have remained roughly as estimated here. However, in the pandemic year April 2020–March 2021, uptake fell from around 70% to 61.8%, so the overdiagnosis rate per person invited will be correspondingly smaller for that year.^23^ It should also be noted that our estimates pertain to a programme which offers mammography every 3 years from ages 50–70. A more intensive programme might engender more overdiagnosis, and one would expect a programme with a higher upper age limit to be characterised by higher rates of overdiagnosis.^24^

One strength of this study is that we had access to individual‐level screening and cancer incidence data, and as this was an England wide study, we were able to match the controls on date of birth and geographical district, minimising the possibility that there were differences in underlying breast cancer incidence based on age or region. Limitations of this study include its observational nature, which may entail potential biases, including self‐selection bias. Where the endpoint is incidence of breast cancer, this bias tends to be conservative, as women attending for screening have been observed to have higher incidence of breast cancer than women who choose not to attend.^13^ We estimated that a modest correction for self‐selection was sufficient. This correction may be too simple, assuming for example a constant relative effect over time. However, the fact that the long‐term odds ratios are close to unity suggests that a more complex correction would still be likely to be modest.

In conclusion, our results showed little if any overdiagnosis, and it is reasonable to conclude that NHSBSP is associated with at worst modest overdiagnosis of breast cancer.

## AUTHOR CONTRIBUTIONS

Oleg Blyuss and Amanda Dibden carried out statistical analysis and initial drafting. Nathalie J Massat was responsible for protocol development and study conduct. Dharmishta Parmar was responsible for informatics and data management. Jack Cuzick, Stephen W Duffy, and Peter Sasieni were responsible for study concept. All authors contributed to the final drafting.

## FUNDING INFORMATION

This research was funded by the National Institute for Health Research (NIHR) Policy Research Programme, conducted through the Policy Research Unit in Cancer Awareness, Screening, and Early Diagnosis, PR‐PRU‐1217‐21601. The views expressed are those of the authors and not necessarily those of the NIHR or the Department of Health and Social Care.

## CONFLICT OF INTEREST

Professor Peter Sasieni has received personal fees from GRAIL Bio outside the submitted work. The other authors report no conflict of interest.

## ETHICS

The study protocol was reviewed and approved by the Department of Health. Ethical approval was obtained from the London Research Ethics Committee of the National Research Ethics Service (reference: 12/LO/1041), and by the National Information Governance Board Ethics and Confidentiality Committee (reference: ECC 6–05 [e]/2012).

## Supporting information


Table S1

## Data Availability

The data that support the findings of this study are available on request from the corresponding author. The data are not publicly available due to privacy or ethical restrictions.
