# Supplementary material for: A case–control study to evaluate the impact of the breast screening programme on breast cancer incidence in England
Source: Cancer Med. 2022 Jul 18;12(2):1878–87. doi: 10.1002/cam4.5004 (PMC9883434; doi:10.1002/cam4.5004)
Supplement: Supplementary file 1 — Table S1 [file CAM4-12-1878-s001.docx]

**Supplementary Table 1.** Odds ratios by age and time since last screen

1. Uncorrected odds ratios

| Age | Time since last screen (years) | | | | | | | | | |
| --- | --- | --- | --- | --- | --- | --- | --- | --- | --- | --- |
|  | 0-<1 | 1-<2 | 2-<3 | 3-<4 | 4-<5 | 5-<6 | 6-<7 | 7-<8 | 8-<9 | 9+ |
| All | 2.46 | 0.42 | 0.54 | 0.85 | 0.76 | 0.88 | 1.01 | 0.98 | 1.04 | 1.05 |
| 47-59 | 2.55 | 0.50 | 0.63 | 0.89 | 0.93 | 0.94 | 1.13 | 0.94 | 1.54 | 1.14 |
| 60-64 | 2.68 | 0.43 | 0.55 | 0.92 | 0.87 | 1.05 | 1.06 | 1.07 | 0.98 | 1.16 |
| 65-69 | 2.60 | 0.37 | 0.50 | 1.02 | 0.85 | 0.79 | 1.26 | 1.30 | 1.19 | 1.16 |
| 70+ | 1.82 | 0.30 | 0.46 | 0.68 | 0.59 | 0.75 | 0.83 | 0.80 | 0.85 | 0.93 |
| 47-69 | 2.61 | 0.44 | 0.57 | 0.94 | 0.90 | 0.93 | 1.15 | 1.13 | 1.16 | 1.19 |
| All | 2.46 | 0.42 | 0.54 | 0.85 | 0.76 | 0.88 | 1.01 | 0.98 | 1.04 | 1.05 |

1. Odds ratios corrected for self-selection

| Age | Time since last screen (years) | | | | | | | | | |
| --- | --- | --- | --- | --- | --- | --- | --- | --- | --- | --- |
|  | 0-<1 | 1-<2 | 2-<3 | 3-<4 | 4-<5 | 5-<6 | 6-<7 | 7-<8 | 8-<9 | 9+ |
| All | 2.34 | 0.40 | 0.51 | 0.81 | 0.72 | 0.84 | 0.96 | 0.93 | 0.99 | 1.00 |
| 47-59 | 2.43 | 0.48 | 0.60 | 0.85 | 0.89 | 0.90 | 1.08 | 0.90 | 1.47 | 1.09 |
| 60-64 | 2.55 | 0.41 | 0.52 | 0.88 | 0.83 | 1.00 | 1.01 | 1.02 | 0.93 | 1.10 |
| 65-69 | 2.48 | 0.35 | 0.48 | 0.97 | 0.81 | 0.75 | 1.20 | 1.24 | 1.13 | 1.10 |
| 70+ | 1.73 | 0.29 | 0.44 | 0.65 | 0.56 | 0.71 | 0.79 | 0.76 | 0.81 | 0.89 |
| 47-69 | 2.49 | 0.42 | 0.54 | 0.90 | 0.86 | 0.89 | 1.10 | 1.08 | 1.10 | 1.13 |
| All | 2.34 | 0.40 | 0.51 | 0.81 | 0.72 | 0.84 | 0.96 | 0.93 | 0.99 | 1.00 |
